# Data mining tools for *Salmonella *characterization: application to gel-based fingerprinting analysis

**DOI:** 10.1186/1471-2105-14-S14-S15

**Published:** 2013-10-09

**Authors:** Wen Zou, Hailin Tang, Weizhong Zhao, Joe Meehan, Steven L Foley, Wei-Jiun Lin, Hung-Chia Chen, Hong Fang, Rajesh Nayak, James J Chen

**Affiliations:** 1 Division of Bioinformatics and Biostatistics, U.S. Food and Drug Administration, Jefferson, Arkansas, USA; 2 Division of Microbiology, U.S. Food and Drug Administration, Jefferson, Arkansas, USA; 3 Department of Applied Mathematics, Feng Chia University, Taichung, Taiwan; 4 Graduate Institute of Biostatistics and Biostatistics Center, China Medical University, Taichung, Taiwan; 5 The Office of Scientific Coordination, National Center for Toxicological Research, U.S. Food and Drug Administration, Jefferson, Arkansas, USA

**Keywords:** Data mining, Salmonella, PFGE, bioinformatics tools, data analysis.

## Abstract

**Background:**

Pulsed field gel electrophoresis (PFGE) is currently the most widely and routinely used method by the Centers for Disease Control and Prevention (CDC) and state health labs in the United States for *Salmonella *surveillance and outbreak tracking. Major drawbacks of commercially available PFGE analysis programs have been their difficulty in dealing with large datasets and the limited availability of analysis tools. There exists a need to develop new analytical tools for PFGE data mining in order to make full use of valuable data in large surveillance databases.

**Results:**

In this study, a software package was developed consisting of five types of bioinformatics approaches exploring and implementing for the analysis and visualization of PFGE fingerprinting. The approaches include PFGE band standardization, *Salmonella *serotype prediction, hierarchical cluster analysis, distance matrix analysis and two-way hierarchical cluster analysis. PFGE band standardization makes it possible for cross-group large dataset analysis. The *Salmonella *serotype prediction approach allows users to predict serotypes of *Salmonella *isolates based on their PFGE patterns. The hierarchical cluster analysis approach could be used to clarify subtypes and phylogenetic relationships among groups of PFGE patterns. The distance matrix and two-way hierarchical cluster analysis tools allow users to directly visualize the similarities/dissimilarities of any two individual patterns and the inter- and intra-serotype relationships of two or more serotypes, and provide a summary of the overall relationships between user-selected serotypes as well as the distinguishable band markers of these serotypes. The functionalities of these tools were illustrated on PFGE fingerprinting data from PulseNet of CDC.

**Conclusions:**

The bioinformatics approaches included in the software package developed in this study were integrated with the PFGE database to enhance the data mining of PFGE fingerprints. Fast and accurate prediction makes it possible to elucidate *Salmonella *serotype information before conventional serological methods are pursued. The development of bioinformatics tools to distinguish the PFGE markers and serotype specific patterns will enhance PFGE data retrieval, interpretation and serotype identification and will likely accelerate source tracking to identify the *Salmonella *isolates implicated in foodborne diseases.

## Background

Food safety remains an important concern due in part to the globalization of food supply and foodborne illnesses create an important public health burden in the United States. CDC data indicates that nearly 48 million people become ill, 128,000 are hospitalized, and 3,000 die due to foodborne illnesses each year, and non-typhoidal *Salmonella enterica *is one of the leading causes of illnesses among the top 31 known foodborne pathogens [1]. The characteristics of *Salmonella *infections has changed over time, including changes in the frequency of antimicrobial-resistant *Salmonella *subtypes implicated and the frequency of different serotypes among isolates associated with human infections [2].

Multiple phenotypic and genotypic *Salmonella *subtyping methods have been developed to efficiently detect the cases of human salmonellosis [3]. These methods include traditional phenotype-based approaches such as serotyping [4]; genotype-based methods such as Pulsed Field Gel Electrophoresis (PFGE) [3,5]; DNA sequence-based methods including DNA microarray analysis, multi-locus sequence typing (MLST) [6,7], multi-locus variable-number tandem repeat analysis (MLVA) [8,9] and next-generation sequencing (NGS) [10-14]. Each of the subtyping approaches has been applied in *Salmonella *outbreak strain identification and source tracking; however they each have their own strengths and weaknesses in terms of sensitivity, cost, speed, and robustness.

Large amounts of molecular subtyping data have been generated by academia, private companies and government agencies. Along with the development of new technologies, it is anticipated that new analytical methods will be applied more often in combination with the conventional assays to characterize and subtype foodborne isolates, therefore, enhancing the current food safety and regulatory science paradigm [15]. Facing the large amount of emerging data and technologies, one of the major challenges is the data management, storage, analysis and retrieval, and how to build up the connections and communication for data developed by various subtyping methods. Data mining seeks to find new interesting patterns and relationships in huge amounts of data. Data mining involves the bioinformatics approaches that combine biological data using computational tools and statistical methods to analyze, summarize and transform data into useful information to improve food safety. Such a systematic approach facilitates the extraction and correlation of patterns of knowledge that is implicit in the stored databases.

PFGE is currently the most widely and routinely used molecular subtyping method by CDC and state health labs in the US for *Salmonella *surveillance and outbreak investigation [16]. Although PFGE provides less-detailed genetic information than NGS and other DNA sequence-based methods, it has been successfully used for over twenty years to type *Salmonella *from human patients, foods, and food animal sources because of its discriminatory power, low cost and high reproducibility [3,5]. PulseNet (http://www.cdc.gov/pulsenet), the CDC's molecular surveillance network used for foodborne infections, has the largest and most rich *Salmonella *subtyping database in the world, storing more than 350,000 PFGE patterns of more than 500 serotypes since 1996 [17]. Data mining of this valuable database will provide resources to study the ecology, epidemiology, transmission, and evolution of the emerging *Salmonella *serotypes.

Several commercial software applications have been used to analyze PFGE data, such as BioNumerics (Applied Maths, Inc., Austin, TX), GelCompar II (Applied Maths, Kortrijk, Belgium) and Fingerprinting II version 3 (Bio-Rad, Hercules, USA). BioNumerics is the default software in PulseNet standard protocol [18,19] and has been widely used in PulseNet participating laboratories and other public health laboratories that perform PFGE subtyping for bacterial foodborne pathogens for surveillance and outbreak investigations. These softwares are currently used to analyze PFGE gel images to generate dendrograms for clustering PFGE patterns from different strains of foodborne pathogens. No other methodologies or commercial tools are applicable on PFGE data except for the cluster analysis, which limits the usage of this subtyping technology in understanding the genetic diversities of foodborne bacteria. In addition, BioNumerics and other software have limitations on dealing with large number of samples (less than 20,000 patterns for Bionumerics), which is an obstacle for meta-analysis of the PFGE data and data mining.

In this study, in order to systematically investigate and characterize PFGE patterns of *Salmonella *isolates, BACPAK knowledgebase was created and systematic approaches assembled to build up a functional software package for PFGE data mining. The approaches include PFGE band standardization, *Salmonella *serotype prediction, hierarchical cluster analysis, distance matrix analysis and two-way hierarchical cluster analysis. The development of this software package and the application of its approaches provide a better understanding of *Salmonella *genetic diversity and epidemiology, and contribute to PFGE-based characterization and surveillance of *Salmonella *isolates in outbreak investigations.

## Implementation

### Bacterial pathogen knowledgebase (BACPAK) construction and PFGE database

An integrated genomic *bac*terial *pa*thogen *k*nowledgebase (BACPAK) is being constructed and housed at the NCTR. As an information system which aims to support the research on foodborne bacterial pathogen detection, characterization and outbreak investigation, BACPAK integrates investigational data from NCTR and other government agencies as well as expert-curated published data on foodborne bacterial pathogens (Table 1). As part of the BACPAK knowledgebase data composition, a total of 45,923 *Xba*I-PFGE patterns of *Salmonella enterica *isolates were collected in the PFGE database established in our previous work [20] (Table 1). These patterns were randomly selected within each of the 32 most frequent serotypes from all the submissions from human sources to PulseNet from 2005 to 2010. The imported gel images were processed and analyzed by BioNumerics software (Applied Maths, Inc., Austin, TX, Version 6.0) according to the PulseNet protocol [19]. The band matching was performed at a trace-to-trace optimization value of 1.56% and a band position tolerance set at 1%.

**Table 1:** The data composition in BACPAK and *Salmonella *PFGE fingerprints database.

BACPAK	
**Antimicribial susceptibility test**	**767**

**Antimicribial resistant gene PCR**	**462**

**Plasmid sequence information**	**34**

**PFGE**	**45,923**

**PFGE Database**	

**Serotypes**	**Number of patterns**

**Agona**	**1954**

**Braenderup**	**2008**

**Enteritidis**	**2338**

**Hadar**	**1981**

**Heidelberg**	**2114**

**I 4, **[5]**,12:i:-**	**2281**

**Infantis**	**2078**

**Javiana**	**2102**

**Mississippi**	**1999**

**Montevideo**	**2041**

**Muenchen**	**1970**

**Newport**	**2005**

**Oranienburg**	**1951**

**Paratyphi B var. L(+) tartrate+**	**2011**

**Poona**	**1956**

**Saintpaul**	**2252**

**Thompson**	**2045**

**Typhi**	**1941**

**Typhimurium**	**2064**

**Typhimurium var. 5-**	**2146**

**Anatum**	**478**

**Bareilly**	**426**

**Berta**	**502**

**Derby**	**393**

**Hartford**	**531**

**Litchfield**	**401**

**Mbandaka**	**432**

**Panama**	**516**

**Paratyphi A**	**135**

**Schwarzengrund**	**225**

**Senftenberg**	**189**

**Stanley**	**460**

### PFGE band standardization

Before analysis with the developed tools, the bands of all the PFGE patterns should be normalized. For example, when using *Salmonella *serotype prediction tool, the bands of tested *Salmonella *isolates should be normalized to band classes stored within the database, which are used in the development of training sets for the prediction tools. To accomplish this band normalization, the NCTR fixed band method [20] was implemented to standardize the band classes for cross-group analysis. In this method, the means of the band sizes of two adjacent bands of the training data was used as the standard to normalize the corresponding bands of each new sample. As an example, assume that the training data have a set of descending bands sized as s1, s2, s3, s4..., and the test sample consists of descending bands of t1, t2, t3, t4... if t1 ≤ (s1+s2)/2, t1 is normalized to s2, and if t1 > (s1+s2)/2, it is adjusted to s1 [21]. A total of 39,830 PFGE patterns were band standardized and stored in the database [21].

### *Salmonella *serotype prediction from PFGE fingerprints

Previous studies have reported two classification algorithms, Random Forest (RF) [22] and Support Vector Machine (SVM) [23], to predict *Salmonella *serotypes based on PFGE fingerprints [21,24]. The scripts of the algorithms were based on the packages "RandomForest" and "e1071" in R (version 2.12.1), respectively. Based on the prediction accuracies, the SVM algorithm was chosen to computerize the scripts as a practical tool for *Salmonella *prediction using PFGE fingerprints. The normalized database consisting of 39,830 patterns from 32 serotypes was used as the default standard and training set [21].

### Hierarchical cluster analysis

The distances of any two of the standardized PFGE patterns were measured and hierarchical cluster analysis was pursued by the complete linkage method using "hcluster package" in R [25]. The scripts were converted to a computational tool provided in BACPAK.

### Distance matrix development and two-way hierarchical cluster analysis

In the approach of distance matrix analysis, scripts were written in R to calculate the Jaccard Distance [26] of PFGE patterns for measuring the dissimilarity of PFGE inter- or intra-serotypes patterns. The color from blue to red indicted the values of the Jaccard Distance ranged from 0 to 1. The scripts were computerized as a tool to identify the differences and relationships among the various *Salmonella *patterns within specific serotypes and among the targeted serotypes.

In the two-way hierarchical cluster analysis, scripts were coded using R to calculate the average proportions of the bands present at every designated band location with values ranging from 0 to 1 to build the characteristic parameters of each target serotype. The hierarchical cluster analysis using the complete linkage was applied based on the dissimilarity measures of any two serotypes calculated by the Euclidean distance [27] of the characteristic parameters. The scripts were implemented to pursue a two-way clustering analysis of the PFGE patterns, in which both serotypes and band locations were clustered according to dissimilarity measures to simultaneously identify the associations between serotypes and band locations.

## Results and discussions

To begin to address the need to develop improved analytical tools for PFGE analysis, a software package consisting of the integrated data mining techniques and the PFGE database was established and stored within NCTR's BACPAK. BACPAK is capturing and storing data including antimicrobial susceptibility data, plasmid sequence data, PCR data on antimicrobial resistance genes and PFGE data (Table 1). The PFGE database consisted of 45,923 semi-randomly selected PFGE patterns submitted to PulseNet from 2005 to 2010 (Table 1) [20]. Based on the statistics of the *Salmonella *Annual Report 2006 [2] and *Salmonella *Annual Summary Tables 2009 from CDC [28], isolates from the 32 serotypes represented in the BACPAK database comprised more than 80% of all *Salmonella *reported over the past 14 years in the US [21].

### The approach for PFGE band standardization

Band normalization is the key point to allow the comparison from different dataset. Since BioNumerics was unstable to handle more than 20,000 PFGE patterns, the implemented NCTR fixed band method was especially useful for large dataset analysis. It showed higher accuracies when used to normalize PFGE bands for *Salmonella *serotype prediction in comparison to the conventional BioNumerics fixed band method [21], and made the meta-analysis available to clarify the inter- and intra-serotypes relationships in a large dataset [20]. In addition, NCTR fixed band method transferred the gel-imaged band class into certain digital parameters in the model, and normalized the bands of future candidates with no necessity to upload and save standard band class in BioNumerics [21].

### The prediction approach for *Salmonella *serotype prediction based on PFGE patterns

The prediction algorithm was developed as described previously to identify *Salmonella *serotypes based on their PFGE patterns [21,24]. In these studies, the NCTR fixed band method coupled to the SVM classification produced the highest average predictive accuracies for serotype determination (96.1%) [21]. Therefore, the SVM algorithm was coupled with NCTR standardization method and turned the R scripts which were implemented as a computational prediction tools installed in BACPAK.

**Figure 1:**
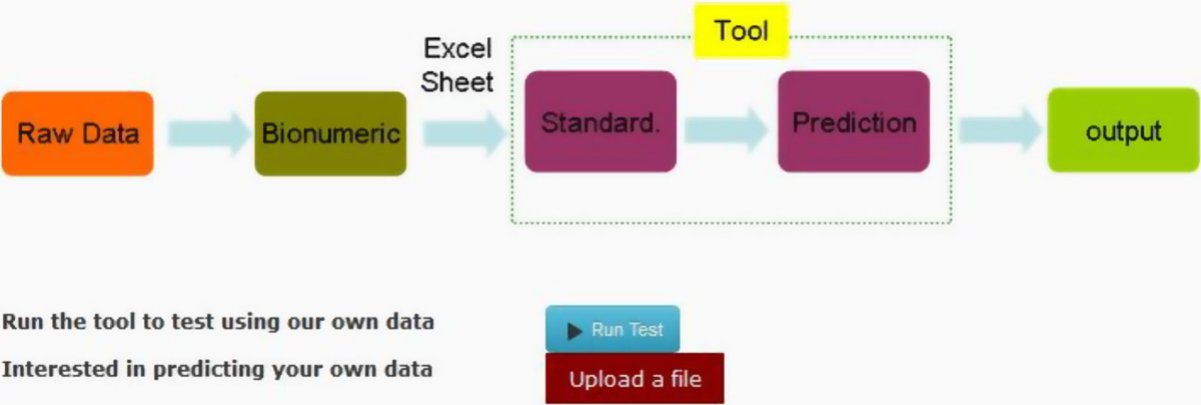
**The flow chart of the tool for *Salmonella *serotype prediction**. (The image was taken from BACPAK.)

Figure 1 shows the work chart of the prediction tool, which includes data normalization, followed by serotype prediction. A total of 39,830 PFGE patterns from the PFGE database were applied as the training dataset bound to the prediction tool [21]. The tool allows individual users to either test the tool with the data in BACPAK or upload their data in a proper format. The output result is presented in an Excel file showing the predicted serotypes.

As a test case, five *Salmonella *isolates (Table 2) were randomly selected from PulseNet but excluded from the training set of 39,830 PFGE patterns. The gel images were processed by BioNumerics software according to the PulseNet protocol [29]. The resultant file containing band presence/absence data was uploaded into the prediction tool in BACPAK. The output result is shown in Table 2. The five tested isolates had their serotypes correctly predicted.

**Table 2:** Five selected test *Salmonella *isolates, the prediction results and the distinguished band markers by the two-way hierarchical cluster analysis tool for five serotype identification ("X" stands for band presence).

Test *Salmonella *isolates	Predicted serotypes	Real serotypes		Distinguished band markers (Kb)								
			**32.8**	**84.9**	**127.2**	**160.1**	**168.3**	**223.2**	**237.5**	**373.9**	**411.8**	**459.9**

**T1: AK_0823200134**	**I 4, **[5]**,12:i:-**	**I 4, **[5]**,12:i:-**		**X**				**X**		**X**		

**T2: AL_AL_8002189-06**	**Thompson**	**Thompson**					**X**					**X**

**T3: CT_02024279**	**Hadar**	**Hadar**		**X**					**X**			

**T4: MD_MD0622721**	**Typhi**	**Typhi**	**X**			**X**						

**T5: TX_TXAML0902385**	**Oranienburg**	**Oranienburg**			**X**						**X**	

The original prediction tools were developed by a supervised classification approach [21,24]. This approach focused on studying the association between PFGE patterns and serotypes determined using traditional serological methods, and applying the information learned from the training set as the rules for prediction in the test set. The prediction accuracy was measured by applying the prediction model based on the training set to emulate the population of the future profiles to be analyzed. If the samples in the training set do not adequately represent the likely samples to be encountered in use, then bias may occur. The training set used in these studies represents greater than 80% of all the isolates reported to CDC, therefore, the prediction tool should be able to predict most *Salmonella *serotypes. As such, this tool should be especially useful to predict the serotype of outbreak isolates before the conventional methods were carried out in a laboratory. The refinement of the predictive tool is an ongoing effort as additional PFGE data becomes available and is incorporated into the training dataset to improve the prediction accuracies.

### Hierarchical cluster analysis

**Figure 2:**
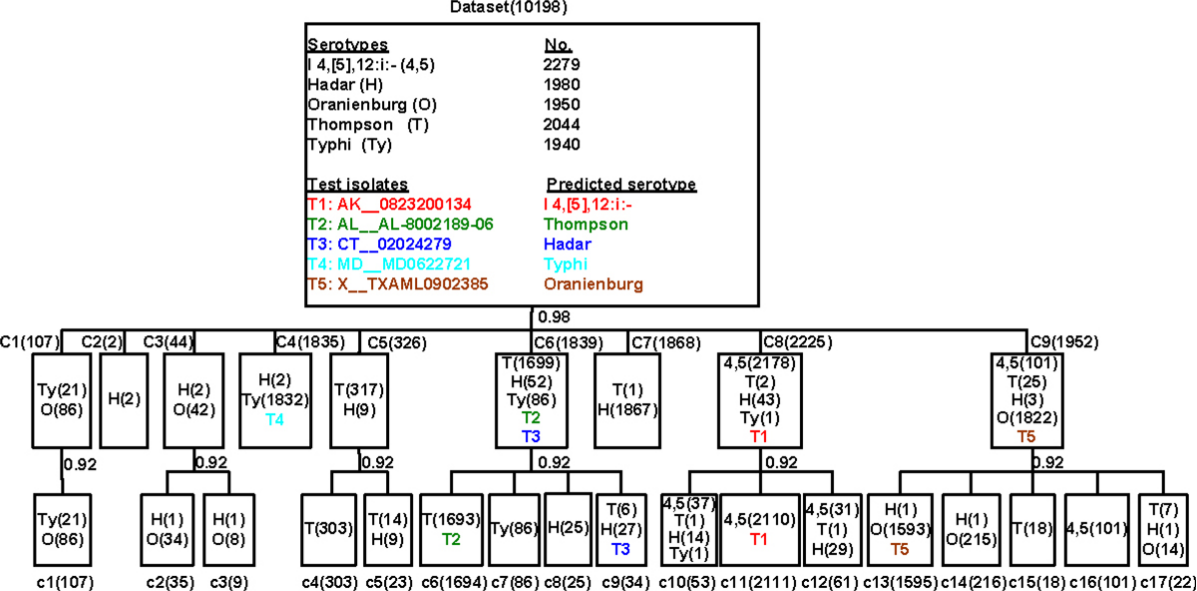
**Hierarchical cluster analysis of PFGE *Xba*I patterns of 10,198 (10,193+5) *Salmonella *isolates**. The dendrogram shows a simplified tree-structure of 10,198 isolates of five serotypes: *S*. I 4, [5],12:i:- (4,5), *S*. Hadar (H), *S*. Oranienburg (O), *S*. Thompson (T), and *S*. Typhi (Ty). Five of 10,198 isolates are test isolates and labeled as T1 to T5 in various colors, while the rest of the isolates are retrieved from the PFGE database bound with the tools. The number in parentheses indicates the number of isolates in the branch squares. There are nine major clusters (C1 to C9) and 17 sub-clusters (c1 to c17) grouped by the hierarchical cluster analysis tool.

Unlike the supervised classification algorithm, hierarchical cluster analysis is unsupervised, where the samples are grouped into subsets based only on the pairwise similarities among their PFGE profiles without using serotype information [24]. As a case study, five test *Salmonella *isolates were added to the dataset of the 10,193 PFGE patterns of the five predicted serotypes (Table 2) which were retrieved from the PFGE database in BACPAK. The hierarchical analysis tool was applied on this dataset (10,198 PFGE patterns) and the dendrogram is shown in Figure 2. At the cutting threshold of 0.98, all 10,198 patterns were grouped into 9 clusters (C1 to C9), and four of five test samples were separated into four individual groups where the predicted serotypes of the four test isolates matched those of the majority samples in the same cluster (Figure 2). The exception was the isolate CT_02024279 whose serotype was correctly predicted as Hadar by the prediction tool (Table 2). Some subtypes were distinguished at this cutting threshold. For example, the predominant cluster of serotype Oranienburg was in C9 which harbored 1822 patterns of Oranienburg, while two subtypes were located in C1 (86 patterns of Oranienburg) and C3 (42 patterns of Oranienburg) (Figure 2). Serotype Thompson was found to have 1 predominant type (C6) and two subtypes (C5 and C9). When the cutting threshold decreased to 0.92, more subtypes were distinguished (c1 to c17). C5 at threshold of 0.98 was further classified into two groups of c4 and c5 at threshold of 0.92, and c4 had a pure composition of 303 Thompson strains. Test *Salmonella *isolates T2 and T3 in C6 was clarified into different groups of c6 (T2) and c9 (T3). At this cutting threshold, the predominant pure group of each of the five serotypes was identified (C4 for Typhi, c6 for Thompson, C7 for Hadar, c11 for I 4, [5],12:i:- and c13 for Oranienburg), and four of five test *Salmonella *isolates (T1, T2, T4 and T5) were clustered together with the predominant groups of their corresponding serotypes, respectively. T3 was mis-classified with 27 isolates of same serotype Hadar in Thompson predominant group C6, indicating that this subgroup of Hadar isolates had close relationship with serotype Thompson. The tool of hierarchical cluster analysis allows users to distinguish the underlying phylogenetic structures or to discover new subtypes between various PFGE patterns. With the emergence of NGS and other sequencing technologies, the subtypes distinguished by hierarchical cluster analysis tool will be clarified in detail.

### Distinguishing serotype relationships: distance matrix and two-way hierarchical cluster analysis

**Figure 3:**
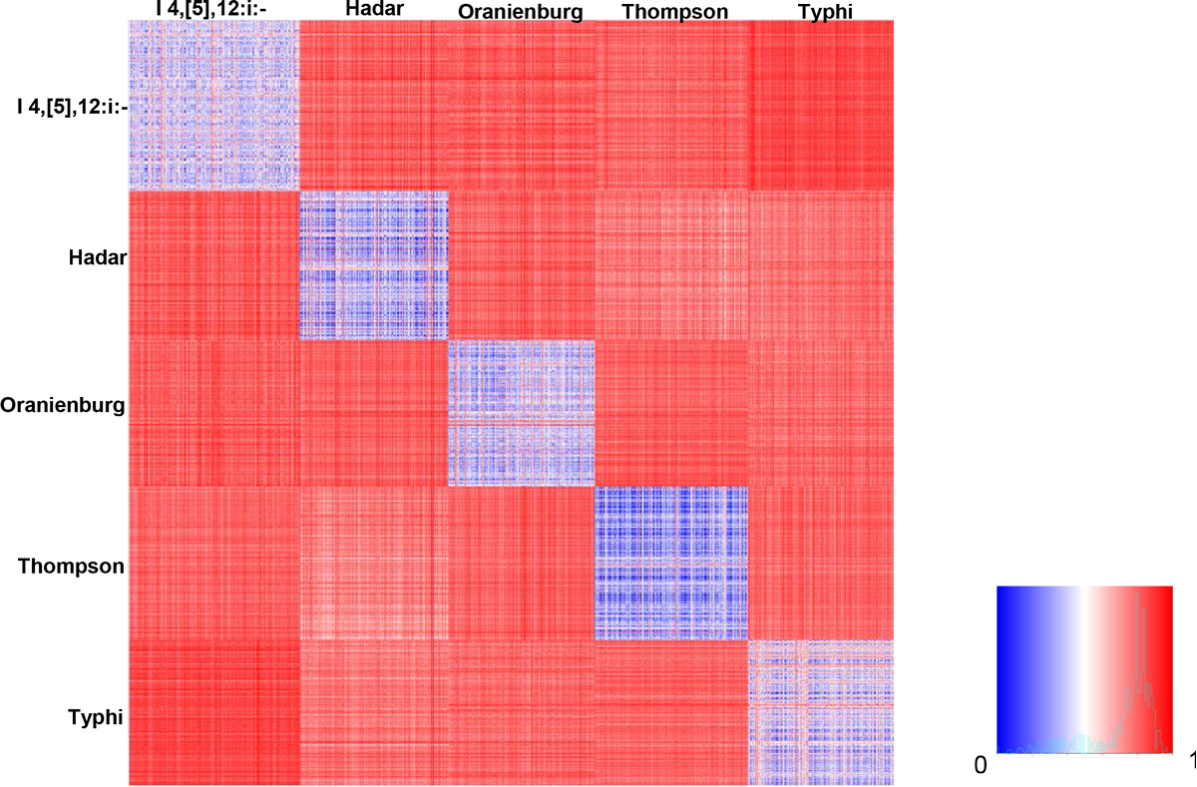
**Distance matrix of five selected serotypes**. The heatmap shows the distances matrix presenting the dissimilarities for any two patterns in the selected dataset of five serotypes. The dissimilarity of PFGE patterns inter- or intra-serotypes was calculated by Jaccard Distance, and the values ranged from 0 (blue) to 1 (red) (shown in the index).

Distance matrix and two-way hierarchical cluster analysis provide users the appropriate methods to further distinguish the relationships among *Salmonella *serotypes. Five serotypes were selected to demonstrate the functionality of these tools. A total of 10,193 PFGE patterns belonging to the five serotypes (Table 2) were retrieved from the database and uploaded to the two tools. Figure 3 exhibited the heatmap of the distance matrix of 10,193 PFGE patterns of five serotypes retrieved from the database as a case study. The squares in the matrix showed various colors ranging from blue to red, indicating various degrees of similarity of patterns within every pair of serotypes. Five blue squares in the diagonal, which were distinguishable from the other squares, represent the close distances between the various patterns within the same serotype (Figure 3). The rest of the squares were red or red/white, indicating the distances between the patterns of their corresponding horizontal and vertical serotypes. The bright blue square of serotype Thompson indicated that 2,045 patterns of this serotype were similar to each other; while serotype Hadar showed close relationship with serotype Thompson and Typhi (pale red/white squares). The result was concordant with the prediction accuracies [21] and hierarchical cluster analysis (Figure 2). Users can directly visualize the similarities/dissimilarities of any two individual patterns and the inter- and intra-serotype relationships of two or more serotypes by using this distance matrix analysis tool with the bound PFGE database.

**Figure 4:**
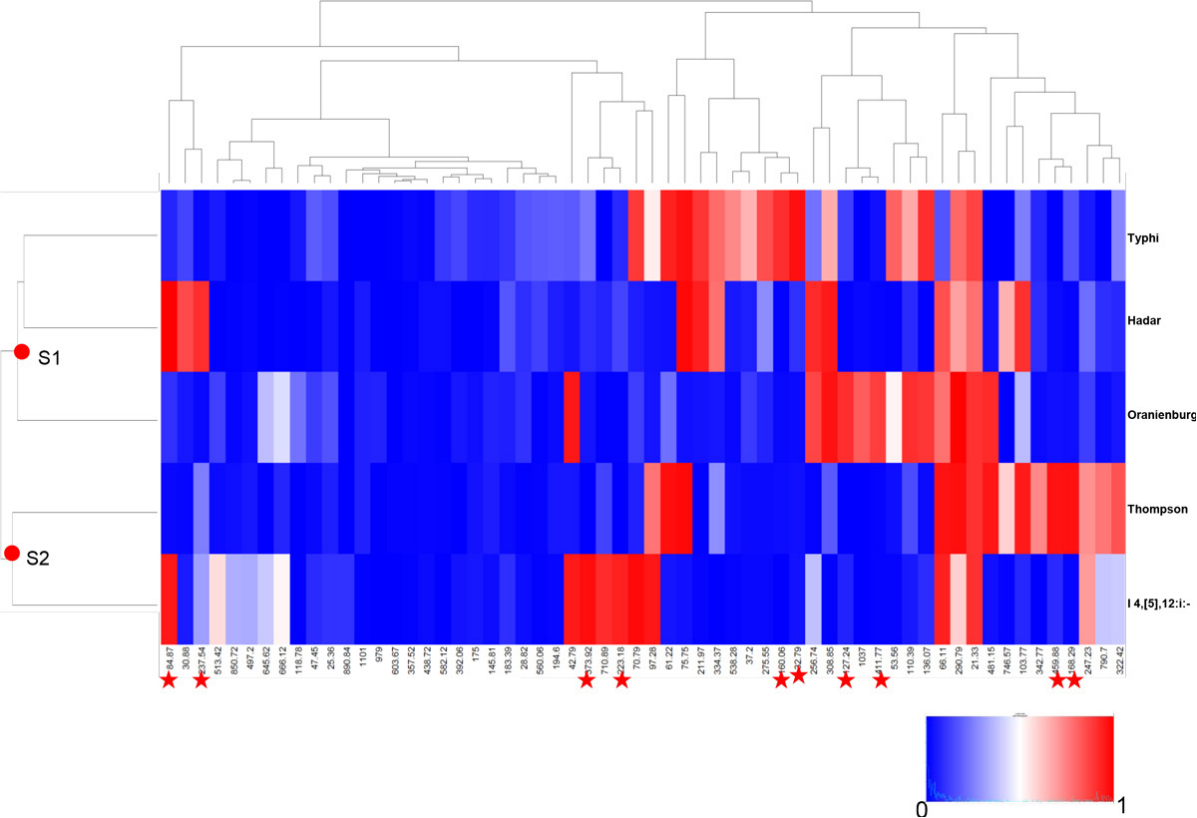
**Two-way hierarchical clustering analysis of the five selected serotypes**. The color histogram shows the proportions of the bands present at every designated band location with values ranging from 0 to 1. The hierarchical cluster analysis was applied based on the dissimilarity measures of any two serotypes calculated by the Euclidean distance of the characteristic parameters. Both serotypes and band locations were clustered according to dissimilarity measures. Red asterisks indicated the distinguished band markers.

The tool of two-way hierarchical cluster analysis provides the summary of the overall relationships between selected serotypes as well as the distinguishable band markers of these serotypes. Using this tool, the hierarchical cluster analysis is applied to the dissimilarity measures of any pair of the selected serotypes calculated by the Euclidean distance of the characteristic parameters. The color of each of the blocks from blue to red represents the various average proportions (between 0 and 1) of band occurrences for each of the selected serotypes (Figure 4). As a case study, the tool was applied to the dataset of 10,193 PFGE patterns of five serotypes retrieved from the database, and the result was shown in Figure 4, where both serotypes and band locations were grouped to simultaneously identify the associations between serotypes and bands. The five serotypes were divided into two groups (S1 and S2). The cluster image indicated a group of marker bands (highlighted with red asterisks) which distinguish the five serotypes (Table 2). The tool shows more advantages in identification of serotype relationships when more serotypes are selected. Our previous research applied this tool to a meta-analysis of 32 serotypes and reported the close relationship of PFGE patterns between serotypes Hadar to Infantis and Muenchen to Newport [20].

The five functional tools were assembled and integrated into a software package to study PFGE profiles for better understanding the genetic diversity of *Salmonella *and other foodbornbe pathogens. The analysis tools included in the package allow the systematic analysis of PFGE data from various aspects and make it available to meta-analyze PFGE profiles from large data sets. The software package is currently available in the NCTR internal BACPAK knowledgebase. BACPAK, as a general-purposed bioinformatics pipeline for foodborne pathogen analysis, will be a new addition to the FDA bioinformatics tools at http://www.fda.gov/ScienceResearch/BioinformaticsTools/default.htm.

## Conclusions

Although NGS and other sequencing technologies are advancing rapidly in foodborne pathogen subtyping, PFGE is still the most widely used method to characterize *Salmonella *strains isolated from outbreaks[30]. In the developed software package, PFGE band standardization normalizes the data for cross-group large dataset analysis. The *Salmonella *serotype prediction tool based on PFGE patterns allows rapid and accurate prediction of *Salmonella *serotypes from outbreaks before the conventional serological methods are pursued. It also shows advantages in distinguishing an isolate that is serotyped as "unknown" by conventional methods, or for a laboratory where standard serotyping is not available. Hierarchical cluster analysis could be used to clarify the subsets of a group of PFGE patterns for source tracking and identification of outbreak isolates. Since *Salmonella *serotypes can be closely related in terms of their virulence, and antimicrobial resistance profiles [17,30-33], our distance matrix analysis and two-way hierarchical analysis tools make it possible to study the relationships between phenotypes and genotypes of *Salmonella *isolates and to distinguish band markers and PFGE pattern diversity for serotype identification, especially for large dataset analysis. Theoretically, these approaches could be applied to other gel-based analysis and other pathogens in the future. Combined with the *Salmonella *genome sequencing data, the distinct serotype specific patterns and bands may provide useful information to aid in the distribution of serotypes in the population and potentially reduce the need for laborious analyses, such as traditional serotyping. In addition, the PFGE analysis tools in the software package are expected to help the *in silico *pattern construction to match PFGE data with NGS data in future studies.

## Competing interests

The authors declare that they have no competing interests.

## Authors' contributions

WL, HC, WZ (Zhao) and WZ performed all calculations and data analysis, and WZ wrote the first draft of manuscript. WZ and JC developed the methods and had the original idea and guided the data analysis and presentation of results. WZ, SF and RN collected and generated data, and HT, JM and HF constructed the database and computerized the scripts into practical tools. All authors contributed in data verification, and approach evaluation and assisted with writing the manuscript. All authors read and approved the final manuscript.
